# Dissecting the Proton Transport Pathway in Oral Squamous Cell Carcinoma: State of the Art and Theranostics Implications

**DOI:** 10.3390/ijms20174222

**Published:** 2019-08-29

**Authors:** Alejandro I. Lorenzo-Pouso, Mario Pérez-Sayáns, Samuel Rodríguez-Zorrilla, Cintia Chamorro-Petronacci, Abel García-García

**Affiliations:** Oral Medicine, Oral Surgery and Implantology Unit, Faculty of Medicine and Odontology, University of Santiago de Compostela, GI-1319 Research Group, Health Research Institute of Santiago de Compostela (IDIS), Santiago de Compostela, 15782, Spain

**Keywords:** mouth neoplasm, carbonic anhydrases, monocarboxylate transporters, Na^+^/H^+^ exchanger, V-ATPase

## Abstract

Cancer cells overexpress proton exchangers at the plasma membrane in order acidify the extracellular matrix and maintain the optimal pH for sustaining cancer growth. Among the families of proton exchangers implicated in carcinogenesis, carbonic anhydrases (CAs), monocarboxylate transporters (MCTs), Na^+^/H^+^ exchangers (NHEs), sodium bicarbonate cotransporters (NBCs), and vacuolar ATPases (V-ATPases) are highlighted. Considerable research has been carried out into the utility of the understanding of these machineries in the diagnosis and prognosis of several solid tumors. In addition, as therapeutic targets, the interference of their functions has contributed to the discovery or optimization of cancer therapies. According to recent reports, the study of these mechanisms seems promising in the particular case of oral squamous cell carcinoma (OSCC). In the present review, the latest advances in these fields are summarized, in particular, the usefulness of proton exchangers as potential prognostic biomarkers and therapeutic targets in OSCC.

## 1. Introduction

**Figure 1:**
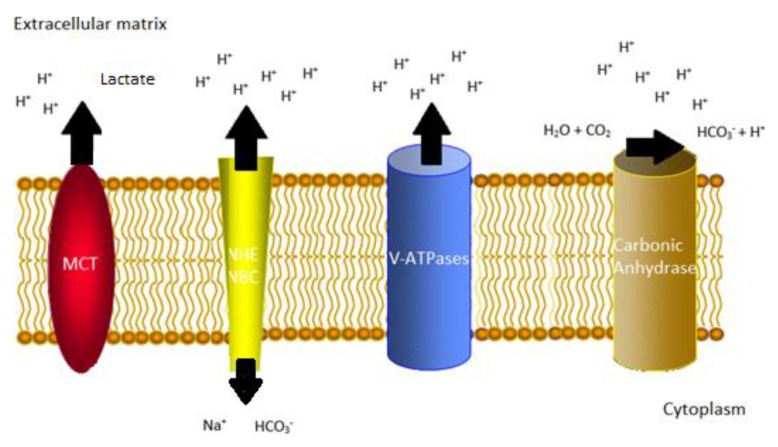
Schematic model of the effects of proton pump regulators on cancer cells. MCT, monocarboxylate transporter; NHE, N^+^/H^+^ transporter; NBC, sodium bicarbonate cotransporter; V-ATPase, vacuolar ATPase.

Otto Warburg first reported on the metabolic phenomenon of many tumor cells, namely, their high anaerobic glycolytic activity, even in the presence of sufficient oxygen [1]. This metabolic pathway appears in contrast to the highly efficient oxidative phosphorylation used by most eukaryotes in order to yield the energy to sustain homeostasis [2]. These changes, both intracellularly and in the extracellular matrix (ECM), are related to cancer progression and metastasis due to their ability to create acid–base disturbances, inducing hypoxia [3]. A common finding across multiple reports examining pH at an ECM level in malignant tissue is its tendency to create an acidic environment; however, this is not the case at the intracellular level, in which the maintenance of metabolic alkalosis is frequently achieved [4]. In the veins, cells have erected barriers against these hazardous events in order to accommodate proton pumping, such as carbonic anhydrases (CAs), monocarboxylate transporters (MCTs), Na^+^/H^+^ exchangers (NHEs), sodium bicarbonate cotransporters (NBCs), and vacuolar ATPases (V-ATPases) [5] (Figure 1). According to several in vivo and in vitro reports and promising I and II phase clinical trials, the study of these pathways for the diagnosis and prognosis of several solid tumors and their interference as therapeutic targets has proven to be a viable avenue for cancer research [6,7]. In particular, drugs which are able to act upon these pathways are able to overcome drug resistance in multidrug resistant cancers. According to governmental agencies, the pleiotropic response of cancer cells to chemotherapy is responsible for the 90% ± 5% outcome failure rates on current therapeutic approaches for solid tumors [8]. At a molecular level, the best characterized multichemoresistance transporters are the adenosine triphosphate-binding cassette superfamily, which is divided into seven subfamilies in which P-glycoprotein (Pgp) and ABCG2 seem to be its pivotal members [9].

Studies which have been carried out show some limitations which hinder their interpretation and are worth mentioning. Most studies have been carried out via cell cultures and studies have considered the proton pumping landscape at a transcriptional and translational level. Nonetheless, most of their regulatory mechanisms are orchestrated by post-translational modifications (PTMs) [10,11].

Oral squamous cell carcinomas (OSCCs) are the most common neoplasia within the head and neck region. In 2012, the estimated global incidence of this cancer per 100,000 person-years was 5.5 in males and 2.5 in females, according to estimates from the GLOBOCAN project [12]. The current OSCC treatment modalities are the same as they have been for the last few decades and these are surgery, radiotherapy, chemotherapy, and immunotherapy. Five-year survival rates for patients with OSCC are greater than 80%, but these survival rates decrease dramatically with the presence of local disseminated metastasis [13,14]. Local relapses of these cancer subtypes are much more frequent than in other head and neck cancers, which also accounts for 24.4% of its death burden [15]. According to a recent bulk of research, proton exchangers are of particular interest in the context of OSCC [16,17]. In fact, our group has performed a series of experiments during the last decade to specifically deal with the implications of CAs, MCTs, and V-ATPases in oral carcinogenesis [16,17]. Despite their diagnostic and therapeutic potential, there are very few studies to date which have directly evaluated the way in which proton transport machinery can contribute towards the onset or progression of OSCC or its contribution to the malignant transition (MT) of oral potentially malignant disorders (OPMDs).

The aim of the present review is to provide the reader with a platform for studying the potential of proton exchangers as diagnostic and therapeutic tools in OSCC. To the best of our knowledge, no review has previously been carried out regarding this nonmainstream approach to dealing with this solid tumor.

## 2. Material and Methods

An electronic search was performed on the following databases: MEDLINE via PubMed, EMBASE, Web of Science, and the Cochrane Library. No publication date, language, or publication status restrictions were applied. Data extraction was performed independently by two of the authors of this review who are experts in oral oncology (A.I.L.-P and M.P.-S) in a pre-defined form. Any discrepancies between the two researchers were resolved by a third author (A.G.G). Different MeSH terms referring to OSCC (cancer of mouth OR cancer of the mouth OR mouth cancer OR mouth neoplasms OR oral neoplasms OR oral cancer OR oral neoplasms) were combined with MeSH terms for each family of proton exchangers (carbonic anhydrases OR monocarboxylic acid transporters OR sodium–hydrogen exchangers OR vacuolar proton-translocating ATPases). Thematic blocks were then constructed on the bases of each proton exchanger family in the form of a literature review. In the case of the proton transport pathway, particular attention was paid to their molecular targets in in vitro and in vivo studies.

## 3. Results

### 3.1. Carbonic Anhydrases

Protons can accumulate in the ECM, not only due to lactate formation, but also as a result of carbonate buffering activity. CAs are a family of metalloenzymes that catalyze the reversible hydration of CO2 to HCO_3_^−^ and H^+^. Sixteen α-CA isoforms have been identified in mammals. This family is subdivided into cytosolic (CAI, CAII, CAIII, CAVII, CAXIII), membrane bound (CAIV, CAIX, CAXII, CAXIV, CAXV), and mitochondrial (CAVA, CAVB) isoforms [18]. Despite the fact that more than 50 genes may be induced by hypoxia via hypoxia-inducible factor 1α (HIF-1α), genes of the *CAIX* isoform seem to be particularly responsive [19]. Also, at a transcriptional level, CAIX has a distinctive proteoglycan fragment which is close to its activation checkpoint and which results in the highest catalytic activity among all membrane-bound CAs [20,21]. CAIX has been used as a therapeutic target in oncology by several selective pharmaceuticals, such as low molecular weight sulfonamide/sulfamate and coumarin derivatives [18].

#### 3.1.1. Diagnostic and Prognostic Implications of Carbonic Anhydrases in Oral Squamous Cell Carcinoma and Oral Potential Malignant Disorders

Only one meta-analysis regarding CAIX immunohistochemical (IHC) expression in head and neck cancers (HNCs) has been conducted to date [22]. Peridis et al. showed that CAIX positivity correlates with poor overall survival (OS) (HR: 1.93; 95%CI 1.41–2.64; *p* < 0.001) as well as disease-free survival (DFS) (HR: 1.77; 95%CI 1.27–2.48; *p* = 0.0008) [22]. Several immunohistochemistry (IHC) reports have dealt with CAIX expression and prognosis in OSCC [17,23,24,25,26,27,28,29,30,31,32,33]. Our group had previously reviewed these reports in a comprehensive review and an update on these IHC-based reports is presented in Table 1 [20]. In this vein, CAIX IHC-based overexpression in OSCC has been frequently correlated with parameters such as lymph node involvement, larger tumor size, advanced clinical stage, poor differentiation, lack of response to treatment, or poorer long-term outcomes (i.e., DFS and OS) [20]. In the biggest cohort published to date, Yang et al. reported that CAIX-positive tumors tended to be at a more advanced stage (*p* = 0.030) and present a higher degree of spread to regional lymph nodes (*p* = 0.026). Likewise, a univariate analysis using the Cox proportional hazard regression model demonstrated that positive CAIX expression is an indicator of poor OS in OSCC (HR: 1.76; 95%CI 1.07–2.87; *p* = 0.025) [32]. In this vein, the two reports published by our group also detected correlation between CAIX expression and N stage and poorer OS [17,29]. Some studies briefly consider CAIX co-expression with other IHC-based markers. Klimowicz et al. proposed the term ‘functional hypoxia’ in order to denote the co-expression derived from the simultaneous immunostaining of Ki-67 and CAIX [34]. These authors found a poorer survival in OSCC patients with combined high CAIX and low tumoral Ki-67, although these authors failed to replicate this observation in a larger independent cohort of patients with OSCC [35]. Kondo et al. also determined the lack of association and prognostic implications of these markers [27]. The expression of CAIX is linked to the expression of a constellation of proteins involved in angiogenesis, such as PAI-1 and VEGFA [33]. Low expression of both CAIX and of HIF-1α was associated with the better long-term outcomes for OSCC [26].

A few studies have characterized the IHC-based expression of CAIX in OPMDs. Our group has focused its efforts on oral leukoplakia (OL) [36]. In our IHC report regarding OL, all samples without dysplasia were negative for CAIX; nonetheless, in cases of positive dysplasia, a wide range of CAIX expression was detected [36]. Zhang et al. recently constructed a nomogram for risk prediction of MT in OL revealing that the combination of P53 and CAIX with some clinical factors (i.e., age and degree of dysplasia) achieved a high prediction accuracy, sensitivity, and specificity (0.96, 0.82, and 0.98, respectively) to predict this outcome [37]. Yang et al. suggested that determining plasma levels of CAIX may be used as a noninvasive technique for monitoring the MT of oral submucous fibrosis [38].

At a transcriptional level, Eckert et al. showed that higher *CAIX* mRNA levels of OSCC tumor cells using a 70% cutoff point were associated with an increased risk of death according to multivariate Cox’s regression hazard analysis (HR: 2.2; 95% CI 1.13–4.36; *p* = 0.02) [39], and these results corroborate our previous report [40]. At a genetic level, Chien et al. reported that *CAIX* gene polymorphisms (i.e., haplotype of *rs2071676*, *rs3829078*, and *rs1048638*) as well as well-characterized OSCC risk factors alter oral cancer susceptibility [41].

In relation to the significance of other CAs in OSCC, a correlation between positive CAI and CAII staining and more advanced clinical stages was reported in addition to larger tumor sizes, although these CAs do not seem to affect long-term outcomes [42].

#### 3.1.2. Carbonic Anhydrases as a Therapeutic Target in Oral Squamous Cell Carcinoma

Limited literature is available regarding the use of these compounds as a therapy or adjuvant for OSCC, and most of this research is based on in vitro experiments. As of now, there is no FDA-recognized adjuvant therapy for this outcome [6]. In this vein, several compounds, especially derivatives of β-amino carbonyl and pyrazoline benzensulfonamides, have been considered as in vitro cytotoxicity-enhancing drug candidates and also CA inhibitor candidates in OSCC cell lines (i.e., Ca9-22, HSC-2, HSC-3, and HSC-4) [43,44,45].

In terms of contributing towards the regulation of multidrug resistance (MDR) of OSCC, Bhattacharya et al. showed the limited usefulness of chemotherapeutic drugs in an OSSC xenograft positive for CAIX, specifically resistance to irinotecan therapy [46]. This finding was later replicated by Chintala et al. [47]. CAIX has been shown to be upregulated in the drug-resistant tongue cancer cell line Tca8113/PYM [48]. Recently Zheng et al. discovered that the *ZEB1–CAIX* signaling axis alterations could partially explain this MDR machinery [49].

### 3.2. Monocarboxylate Transporters

MCTs are a family of 14 proton-linked plasma membrane transporters that carry out lactate and proton exchange passively [50]. In particular, MCT1 and MCT4 have been described as key factors in carcinogenesis. The MCT1 and 4 isoforms are broadly expressed in cancers and have recently been associated with OSCC aggressiveness and prognosis [51]. In a landmark document, Curry et al. postulated that populations of OSCC cells MCT4+ and Ki67− can provide high-energy mitochondrial “fuels” for proliferative cancer cells to burn [52].

#### 3.2.1. Diagnostic and Prognostic Implications of Monocarboxylate Transporters in Oral Squamous Cell Carcinoma

Table 2 presents the IHC reports that have studied this issue. In our cohort, there was no association between OS, DFS, or any clinical or pathological parameter and the presence or absence of any MCT. Nonetheless, the presence of both MCT1 and MCT4 combined with the absence of MCT2 was significantly associated with shorter OS (HR: 95%CI 2.152 1.07–l4.33; *p* = 0.032) [17]. On the contrary, Zhu et al. found that MCT4 overexpression was associated with tumor size, TNM classification, lymphatic metastasis, distant metastasis, and tumor recurrence. They also determined that MCT4 expression was associated with a poorer OS (HR: 3.64; 95%CI 1.59–8.29; *p* = 0.003) and DFS (HR: 3.423 95%CI 1.51–7.78; *p* = 0.002) [53]. Further studies with large samples are warranted to confirm these findings.

#### 3.2.2. Monocarboxylate Transporters as a Therapeutic Target in Oral Squamous Cell Carcinoma

Inhibition of tumor lactate oxidation via MCT targeting has been poorly explored in head and neck tumors. In fact, only a few studies evaluating in vivo and in vitro models are available. Busk et al. demonstrated that using the MCT1 inhibitor α-cyano-hydroxycinnamate in mice bearing FaDu(DD) tumors caused a transient reduction in the Pasteur effect, which can switch tumor oxygenation, indirectly killing its radioresistant feature [55]. Tassone el al. showed that metformin administration on a mouse model of HNC decreased the size of CAL27 xenograft tumors by 45% and reduced MCT1 staining by 28% [56]. Mehibel et al. showed that the combination of simvastatin with AZD3965 (MCT1 inhibitor) led to further tumor growth delay in mice bearing FaDu(DD) xenografts [57]. In this line, Lebo et al. showed that statin use at the time of diagnosis of HPV-negative HNCs was associated with better OS [58].

### 3.3. Na^+^/H^+^ Exchangers and Sodium Bicarbonate Cotransporters

NHEs are membrane proteins that transport Na^+^ into the cell and H^+^ out of the cytoplasm. Although eleven NHE isoforms have been identified in mammals, the most relevant NHE isoform for migrating cells is NHE1, a ubiquitously expressed housekeeping protein involved in the maintenance of the pH and cell volume. NBCs act as a symporter of HCO_3_^−^ and Na^+^ across cell membranes. This effect results in variations in proton-pumping machineries, which has proven to have relevant implications in multiple cancers [59]. Lee et al. demonstrated that several isoforms of these two families of acid extruders NHEs (NHE1) and NBCs (NBCn1, NBCe1, and NDCBE) can promote cytosolic pH recovery in OEC-M1 cells. Nonetheless, they also noticed that ethanol can dismantle these protective functions [60]. Lv et al. showed that blocking NHE1 suppressed the invasion and migration in a human tongue squamous cell carcinoma cell line (Tca8113) following a hypoxic injury [61]. Kaminota et al. also verified the positive effect of the NHE1 inhibitor on the reduction of the metastasis potential of a HNC cell line (SASL1m). This Japanese group also verified that shRNA-mediated NHE1 knockdown in SASL1m resulted in the same effect [62].

### 3.4. Vacuolar ATPases

V-ATPase is an enzyme which is composed of several subunits that exerts pivotal roles in cellular homeostasis, such as the establishment and maintenance of the acidic pH of endocytic and secretory organelles, and pumping protons into lumen in an ATP-dependent manner. Nonetheless, other noncanonical and poorly understood functions have been attributed to this enzyme, such as cytoskeletal tethering or mammalian target of rapamycin 1 (mTOR1) activation [63,64]. Our group suggested that V-ATPase activity is highly increased in OSCC and that this event could be useful for prognosis and diagnosis, and that it could also be a therapeutic target especially when dealing with MDR [65].

#### 3.4.1. Diagnostic and Prognostic Implications of Vacuolar ATPases in Oral Squamous Cell Carcinoma

Initially, our group identified two genes related to this mechanism, ATP6V0C and ATP6V1C1, by means of genes arrays. Later, ATP6V1C1 was found to be overexpressed in 100% of OSCC samples included by real-time PCR (RT-PCR), thus identifying this gene as a useful diagnostic tool [66]. Pérez Sayáns discovered the value of the ATP6V1C1 expression for the diagnosis of these solid tumor by means of an oral brush cytology technique confronting it to normal oral mucosa (NOM). This was confirmed by a receiver operating characteristic (ROC) curve analysis which showed an area under the curve (AUC) of 0.9476 with 81.25% sensitivity and 93.75% specificity [67]. García-García, by means of IHC, identified that ATPase C1 in OSCC was predominantly cytoplasmic, although there was perinuclear and nuclear expression. This report also found a higher expression of these subunits in OSCC than in NOM [68]. Later, our group used tissue microarrays (TMAs) for the semi-quantitative evaluation of ATPase C, finding high intragroup variability within IHC, and also determining that there was no clinical–pathological correlation with its expression in the studied OSCC cohort [69].

#### 3.4.2. Vacuolar ATPases as a Therapeutic Target in Oral Squamous Cell Carcinoma

Several molecules which are able to inhibit V-ATPases have been identified. The first identified V-ATPase inhibitors were compounds of microbial origin, such as bafilomycins and concanamycins which, in fact, have shown interesting anticancer properties (for a review, please see [70]). Nonetheless, pharmacokinetic challenges hinder the clinical translation of these inhibitors, in particular, its cytoxicity in normal cells [70,71]. Kiyoshima et al. demonstrated that a concanamycin A1 can induce apoptosis in vitro in four OSCC cell lines (MISK81-5, SAS, HSC-4, and SQUU-B) and can also confront MDR [72]. Nilsson et al. demonstrated that reduced lysosomal acidification driven by a decrease in ATP6V1B2 could explain a poor response to cisplatin in several HNC cell lines [73].

Other groups of promising V-ATPase inhibitors in the management of HNCs have been characterized, and these include the family of proton pump inhibitors (PPIs) [74]. Although a biological rationale regarding the positive effect of these in adjuvant therapies in HNCs as well as an ecological study have correlated its intake with a better OS, further research is needed in the form of randomized controlled trials [75].

## 4. Concluding Remarks

The main families of enzymes involved in tumor acidification remain poorly explored in OSCC. Nonetheless, several IHC- and mRNA-based studies have ascertained the usefulness of some of its isoforms as promising diagnostic and prognosis tools for this solid tumor. In the case of its use as a therapeutic target or in the fight against MDR, the available evidence identifies them as a relevant avenue for future research. The exploration of proton exchangers in OSCC could be a cornerstone for the improvement of current therapies.

## Figures and Tables

**Table 1:** CAIX immunohistochemical expression in oral squamous cell carcinoma (modified from Pérez-Sayáns et al. (2012) [20]).

Study	OSCC Cases	Positivity n (%)	Negativity n (%)	Quantification
Kim et al. (2007) [23]	60	38 (63.3)	22 (36.7)	CAIX (−) <10%; CAIX (+) >10%
Choi et al. (2008) [24]	117	54 (46.2) ^a^; 14 (11.9) ^b^	49 (41.9)	CAIX (−) <5%; CAIX (1+) 5–20%; CAIX (2+) >20%
Roh et al. (2009) [25]	43	7 (16.3) ^a^; 2 (4.6) ^b^; 10 (23.6); 2 (4.6) ^c^	17 (39.5)	CAIX (−) 0%; CAIX (1+) 1–10%; CAIX (2+) 11–50%; CAIX (3+) 51–80%; CAIX (4+) 81–100%
Eckert et al. (2010) [26]	80	21 (25) ^a^; 11 (13.8) ^b^; 2 (2.5) ^c^	46 (57.5)	CAIX (−) 1–10%; CAIX (1+) 11–50%; CAIX (2+) 51–80%; CAIX (3+) >80%
Kondo et al. (2011) [27]	107	105 (98.1)	2 (1.9)	CAIX (−) <10%; CAIX (+) >10%
Brockton et al. (2012) [28]	61	16 (26.2)	45 (73.8)	CAIX (−) AQUA score within the lower three quartiles; CAIX (+) AQUA score within the upper quartile*
Pérez-Sayáns et al. (2012) [29]	50	18 (36.0) ^a^; 23 (46.0) ^b^	9 (18.0)	CAIX (−) <10%; CAIX (1+) 10–50%; CAIX (2+) >50%
Zhang et al. (2013) [30]	85	58 (58.2)	27 (41.8)	CAIX (−) <5%; CAIX (+) >5%
Hwa et al. (2015) [31]	25	5 (20.0)	20 (80.0)	CAIX (−) <30%; CAIX (+) >30%
Yang et al. (2015) [32]	271	113 (41.7)	158 (58.3)	NR
Simões-Sousa et al. (2016) [17]	124	78 (62.9)	46 (37.1)	CAIX (−) <50%; CAIX (+) >50%
Peterle et al. (2018) [33]	52	19 (36.5) ^a^; 7 (13.5) ^b^	25 (48.1)	CAIX (−) 0%; CAIX (1+) 1–25%; CAIX (2+) >25%

NR, not reported. ^a^ 1+ positivity; ^b^ 2+ positivity; ^c^ 3+ positivity.

**Table 2:** MCT immunohistochemical expression in oral squamous cell carcinoma.

Study	OSCC Cases	Positivity n (%)	Negativity n (%)	Quantification
Zhu et al. (2014) [53]	99	41 (41.5)	58 (58.5)	MCT4 (−), <5%; MCT4 (1+) 5–10% ^a^; MCT4 (2+) 10–50%; MCT4 (3+), 50–75%; MCT4 (4+) >75% ^b^
Jensen et al. (2015) [54]	30	MCT1: 10 (33.3); 20 (66.6). MCT4: 14 (46.7); 2 (6.6%).	MCT1: 0 (0). MCT4: 15 (50.0).	^c^
Simões-Sousa et al. (2016) [17]	124	MCT1: 120 (93.8). MCT2: 47(52.8). MCT4: 89 (71.2)	MCT1: 8 (6.3). MCT2: 42 (47.2). MCT4: 36 (28.8)	MCT1 (−) <50%; MCT1 (+) >50%. MCT2 (−) <50%; MCT2 (+) >50%. MCT4 (−) <50%; MCT4 (+) >50%

^a^ negativity; ^b^ positivity; ^c^ (…) The intensity of staining was divided into weak, medium, and high expression. Weak staining was defined as tissues in which the staining was the same or less than that seen in normal oral mucosa. High expression was defined as the highest possible expression observed, and medium as a staining in between these two (…).

## Abbreviations

CA	carbonic anhydrase
CI	confidence interval
DFS	disease-free survival
ECM	extracellular matrix
FDA	Food and Drug Administration
HIF	hypoxia-inducible factor
HNC	head and neck cancer
HR	hazard ratio
IHC	immunohistochemistry
MCT	monocarboxylate transporter
MDR	multidrug resistance
MT	malignant transformation
MTOR	mammalian target of rapamycin
NBC	sodium bicarbonate cotransporters
NHE	Na^+^/H^+^ exchanger
OPMD	oral potentially malignant disorders
OSCC	oral squamous cell carcinoma
PPI	proton pump inhibitors
PTM	post-translation modification
Pgp	P-glycoprotein
TMA	tissue microarray
V-ATPase	vacuolar ATPase
